# Supplementary material for: Co-expression Networks From Gene Expression Variability Between Genetically Identical Seedlings Can Reveal Novel Regulatory Relationships
Source: Front Plant Sci. 2020 Dec 15;11:599464. doi: 10.3389/fpls.2020.599464 (PMC7770228; doi:10.3389/fpls.2020.599464)
Supplement: Supplementary Figure 1 — Expression in seedlings of genes in module 1, from the RNA-seq data, with one line per gene. Expression is mean normalized for each gene. [file Data_Sheet_1.pdf]

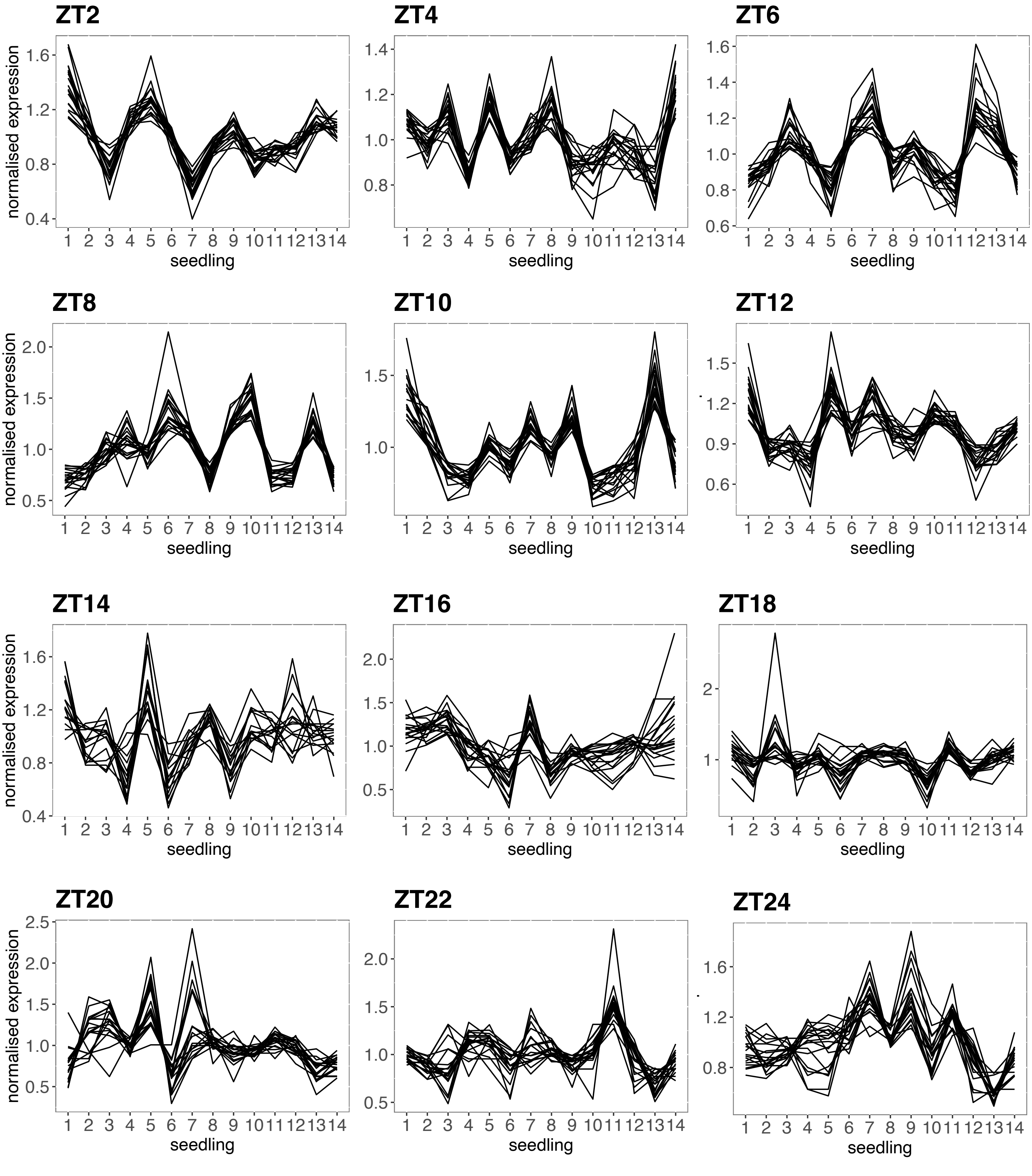

Supplementary figure 1
Expression in seedlings of genes in module 1, from the RNA-seq data, with one line per gene. Expression is mean normalised for each gene.

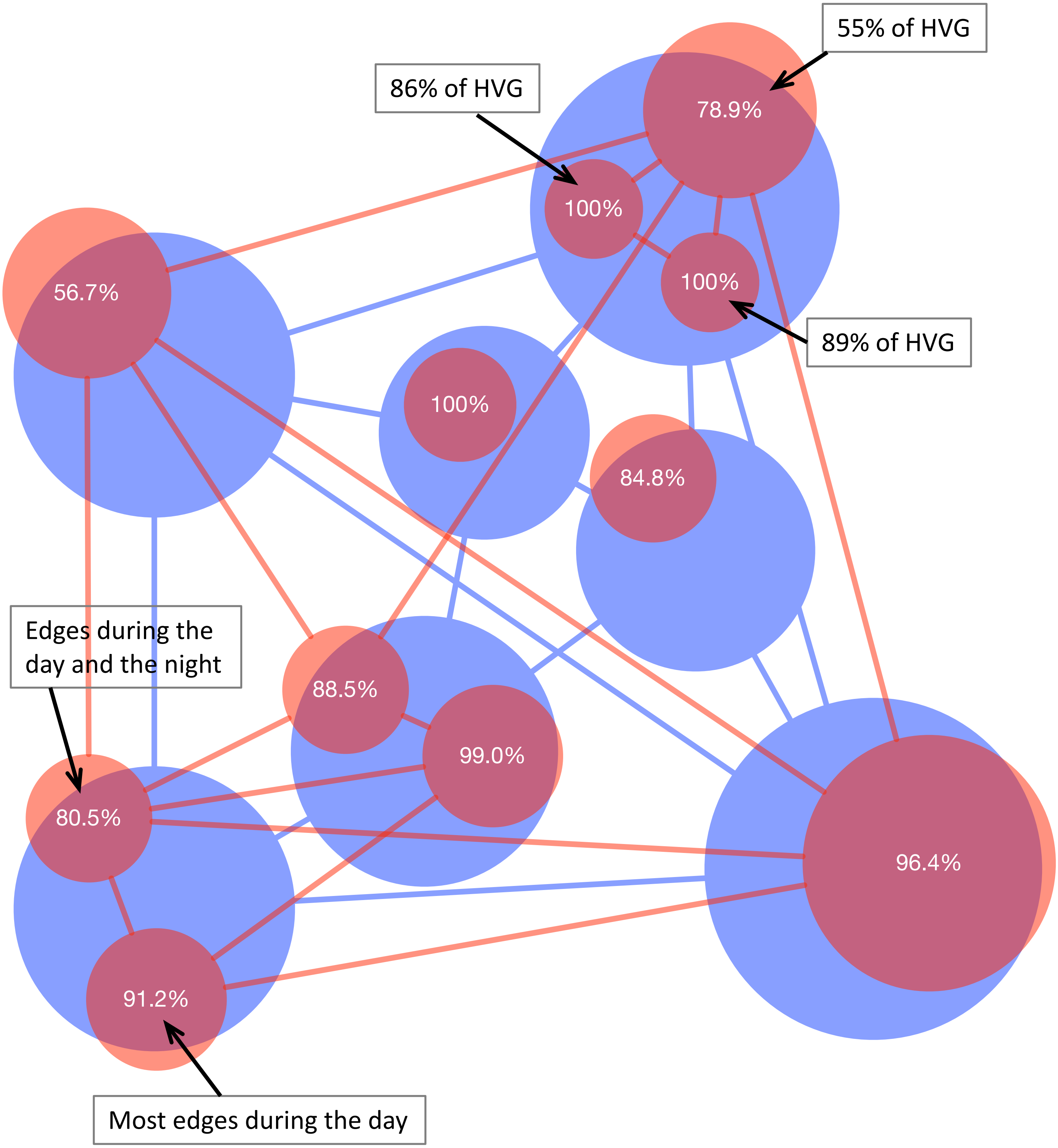

Supplementary figure 2
Comparison of edges in modules detected in the networks containing edges present in 3 or 4 consecutive time points. Modules detected in the network based on edges present in at least 3 consecutive time points are shown in blue. Modules detected in the network based on edges present in at least 3 consecutive time points are shown in red. For the later, the percentage of edges of the modules that are also detected in the blue modules are indicated.

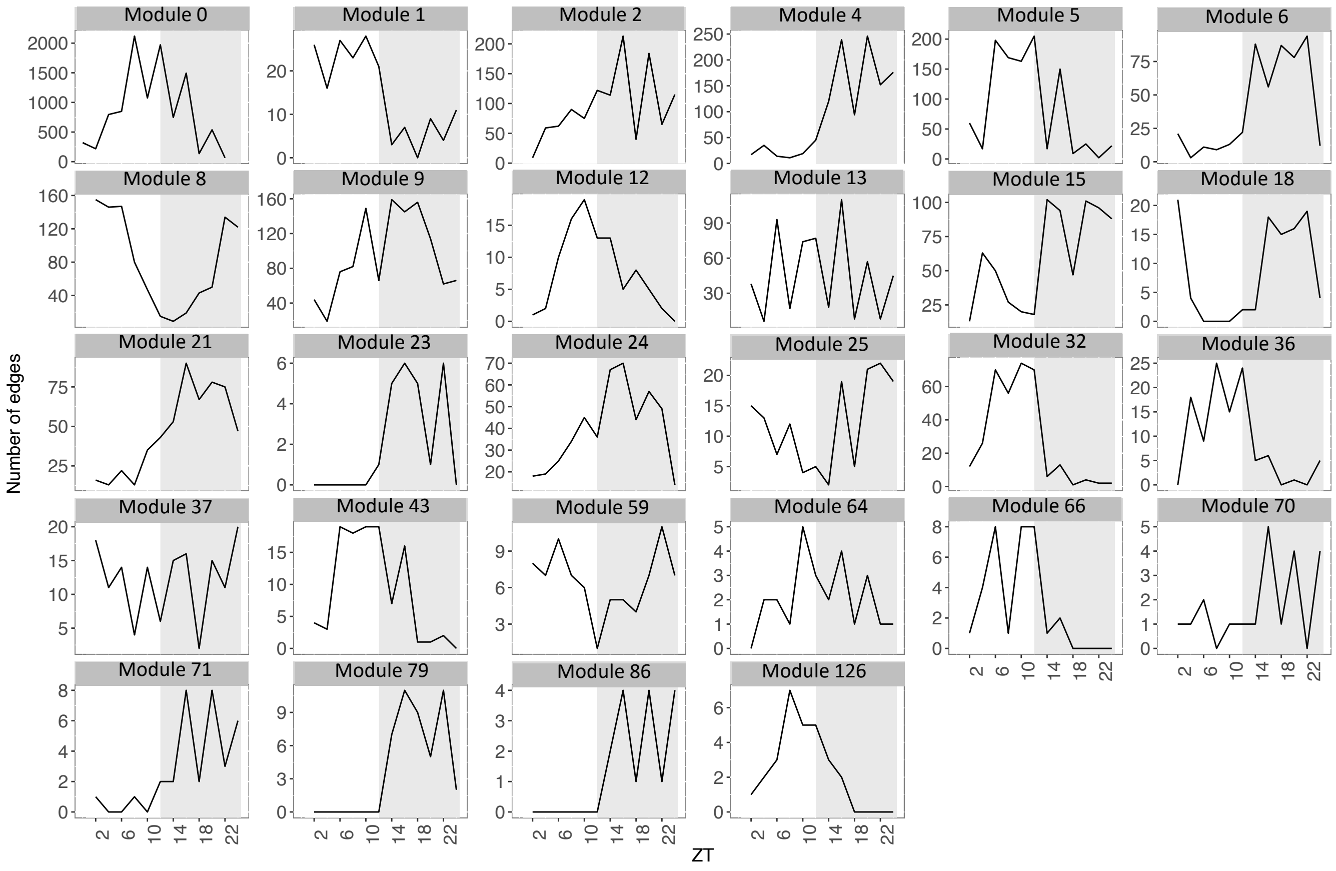

Supplementary figure 3
Number of edges in the final network that are detected in each time point, for every module containing at least 5 genes.

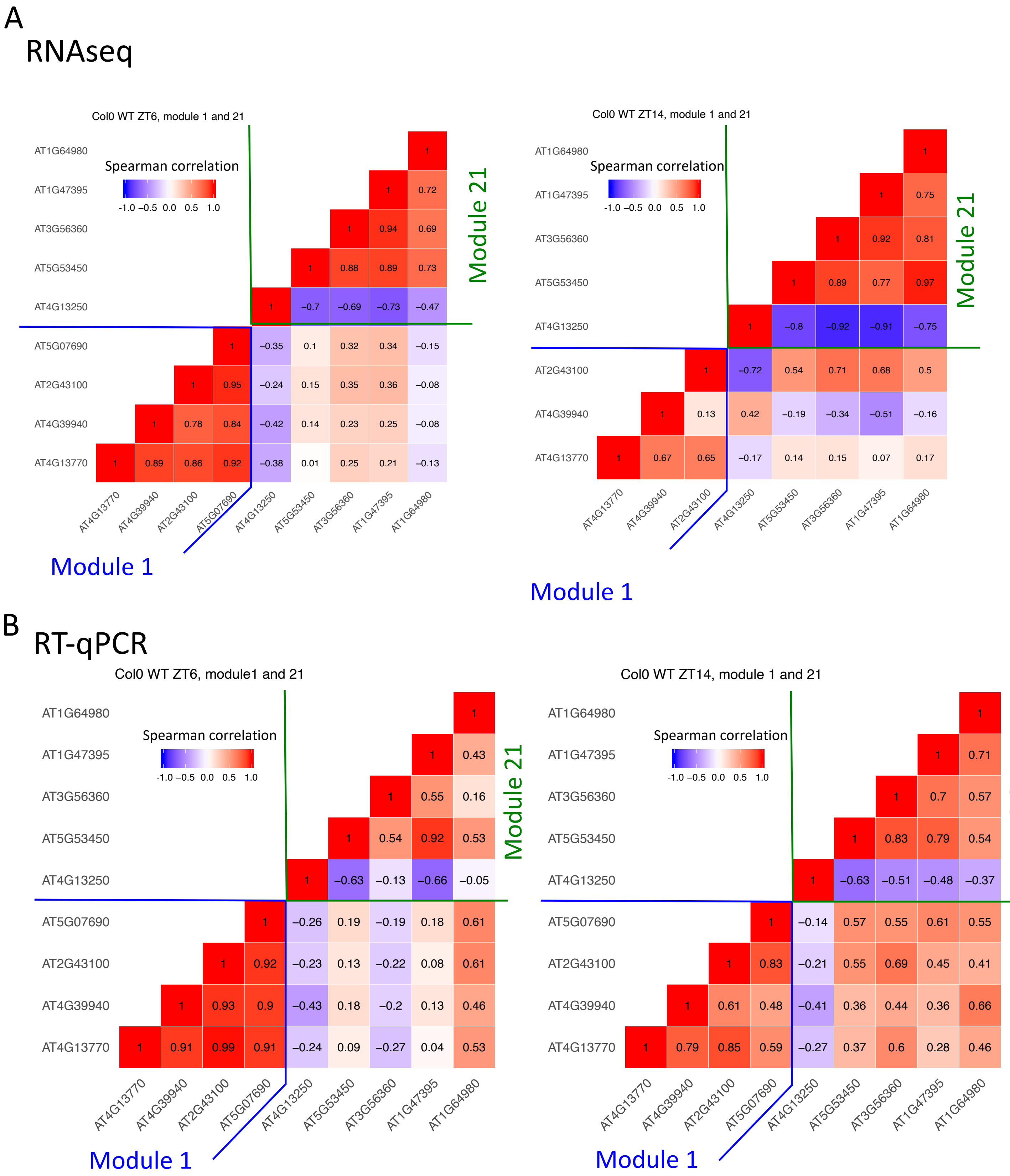
A
RNAseq
Col0 WT ZT6, module 1 and 21
Spearman correlation
-1.0 −0.5 0.0 0.5 1.0
AT1G64980
AT1G47395
AT3G56360
AT5G53450
AT4G13250
AT5G07690
AT2G43100
AT4G39940
AT4G13770
Module 21
Module 1
Col0 WT ZT14, module 1 and 21
Spearman correlation
-1.0 −0.5 0.0 0.5 1.0
AT1G64980
AT1G47395
AT3G56360
AT5G53450
AT4G13250
AT2G43100
AT4G39940
AT4G13770
Module 21
Module 1
B
RT-qPCR
Col0 WT ZT6, module1 and 21
Spearman correlation
-1.0 −0.5 0.0 0.5 1.0
AT1G64980
AT1G47395
AT3G56360
AT5G53450
AT4G13250
AT5G07690
AT2G43100
AT4G39940
AT4G13770
Module 21
Module 1
Col0 WT ZT14, module 1 and 21
Spearman correlation
-1.0 −0.5 0.0 0.5 1.0
AT1G64980
AT1G47395
AT3G56360
AT5G53450
AT4G13250
AT5G07690
AT2G43100
AT4G39940
AT4G13770
Module 1

C

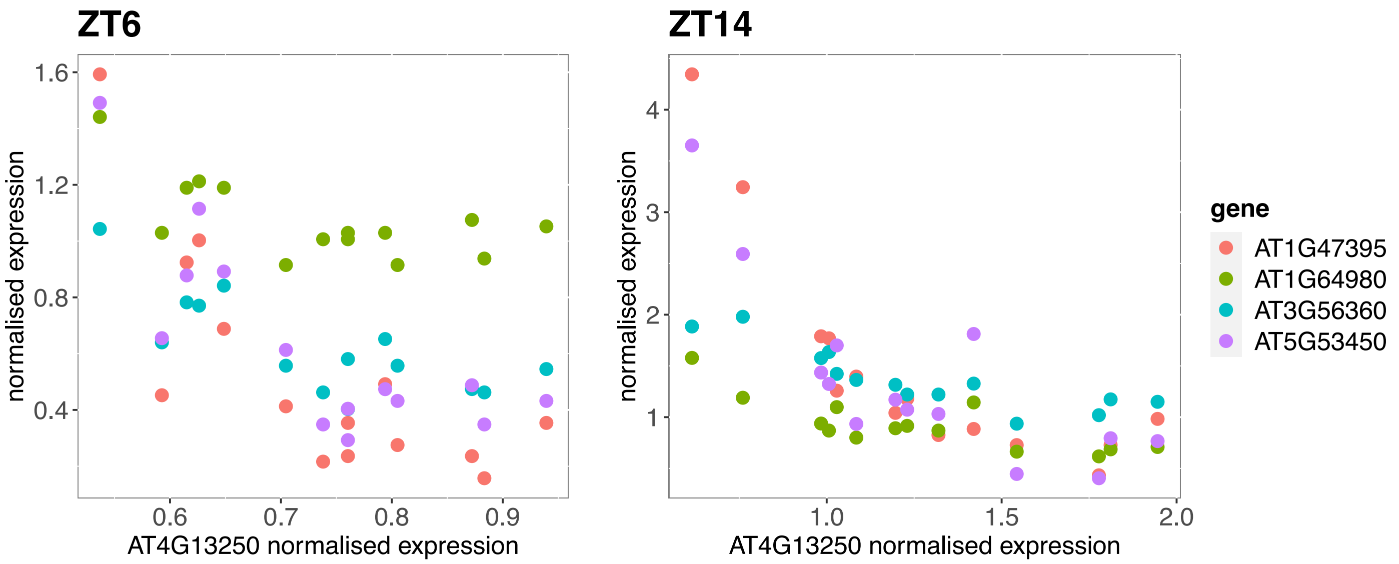

Supplementary figure 4

a. Correlation in expression between seedlings for genes of the module 1 and module 21, for the RNA-seq experiment. AT5G07690 at ZT14 was removed as it is not expressed.

b. Correlation in expression between seedlings for genes of the module 1 and module 21, based on a RT-qPCR replicate of the RNA-seq experiment. Sixteen seedlings where harvested at ZT6 and at ZT14.

c. Normalised expression level in the fourteen seedlings for the genes of the module 21, from the RNA-seq data. Expression level for AT4G13250 is shown as the x axis while expression for the other genes of the module are shown on the y axis. Expression is mean normalised for each gene.

A

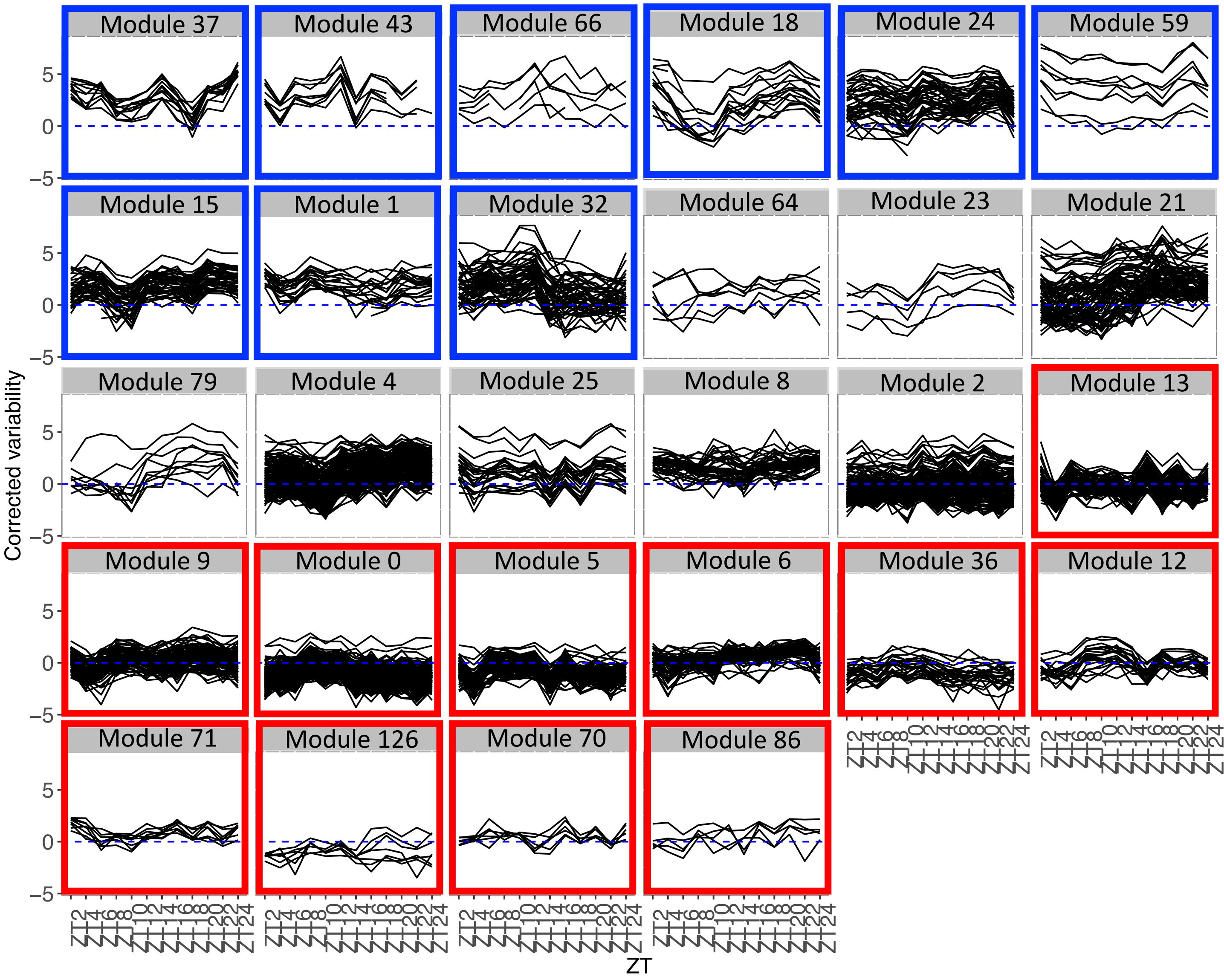

Module 37
Module 43
Module 66
Module 18
Module 24
Module 59
Module 15
Module 1
Module 32
Module 64
Module 23
Module 21
Module 79
Module 4
Module 25
Module 8
Module 2
Module 13
Module 9
Module 0
Module 5
Module 6
Module 36
Module 12
Module 71
Module 126
Module 70
Module 86
Corrected variability
5
0
−5
ZT2
ZT4
ZT6
ZT8
ZT10
ZT12
ZT14
ZT16
ZT18
ZT20
ZT22
ZT24
ZT

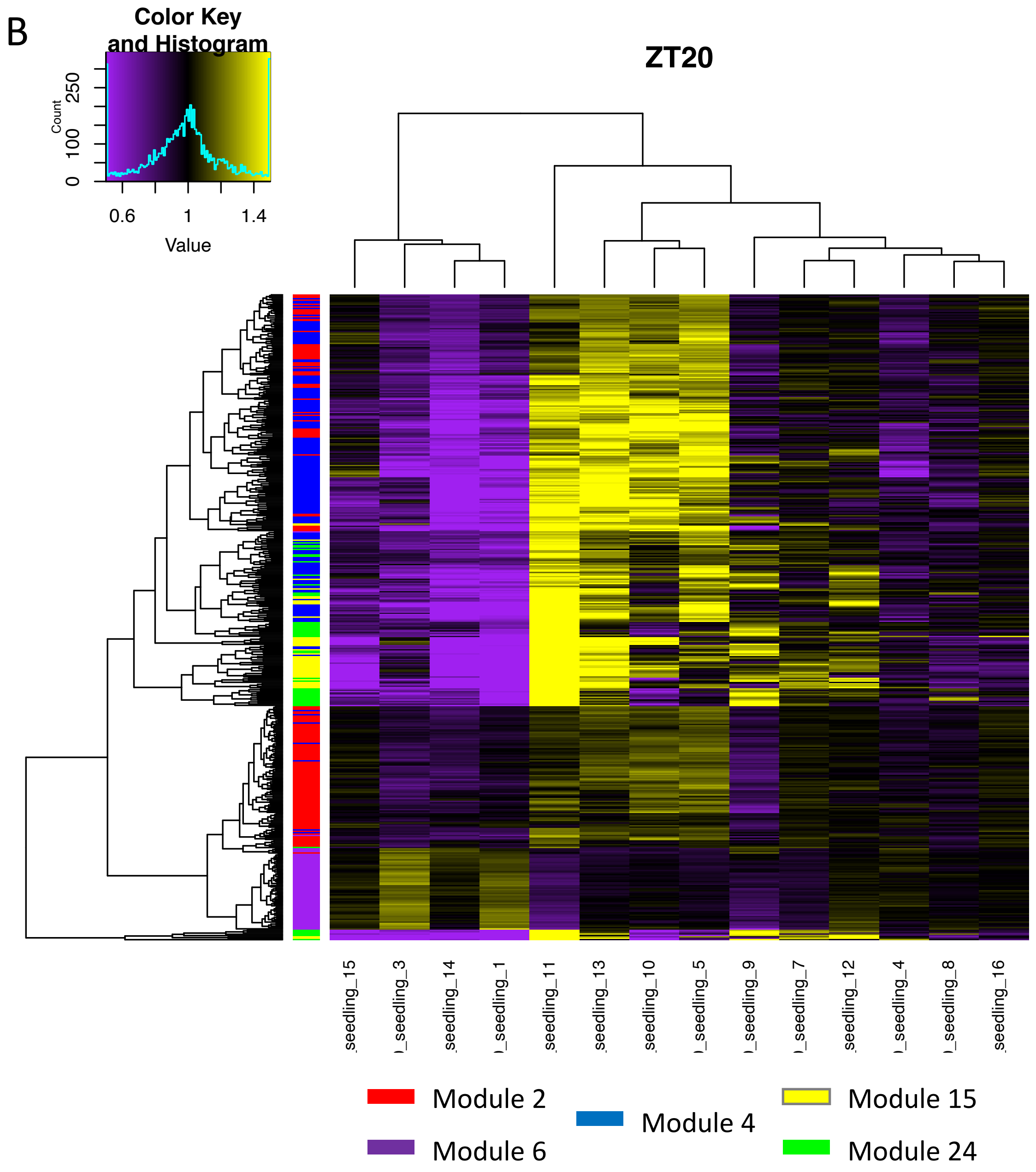

Supplementary figure 5

a. Inter-individual gene expression variability profiles throughout the time course for genes in each module with 5 genes or more. Each line represents the corrected variability level for one gene: : corrected CV^2=[$\log_2(CV^2/\text{trend})$], with CV^2=variance/(average^2)] (see Cortijo et al., 2019).
Modules are ordered by the percentage of HVG (high to low). Modules highlighted in blue contain 75% or more of HVGs. Modules highlighted in red contain 10% or less of HVGs.

b. Heatmap of normalized gene expression for genes in modules 2, 6 (less than 15% HVG), module 4 (55% of HVG) and modules 15 and 24 (more than 85% of HVG). Expression is shown in single seedlings from the time point ZT20. Expression is mean normalised: expression in a seedling/ averaged expression in all seedlings. The left color coded bar indicates the module of each gene.

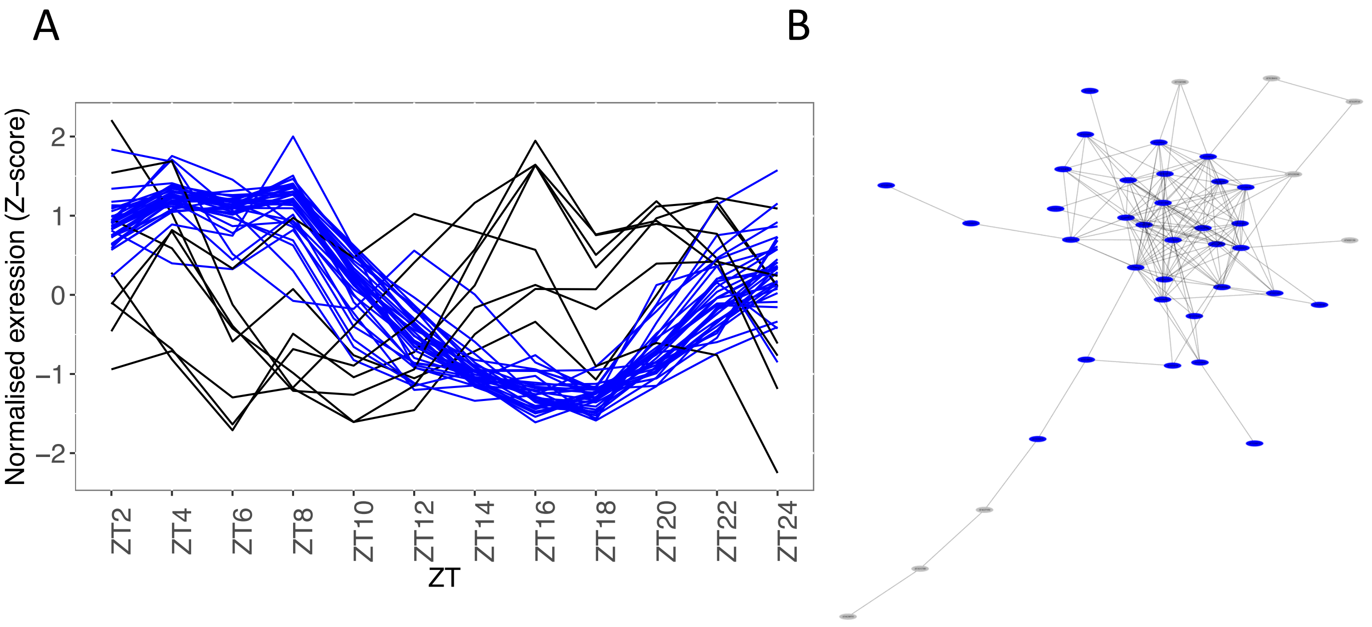

Supplementary figure 6

a. Expression profiles throughout the time course for genes in module 8. Each line represents the normalised expression (z-score) for one gene. Genes of the photosystem I, II or the light harvesting system are in blue.

b. All edges and nodes of module 8. Genes of the photosystem I, II or the light harvesting system are in blue.

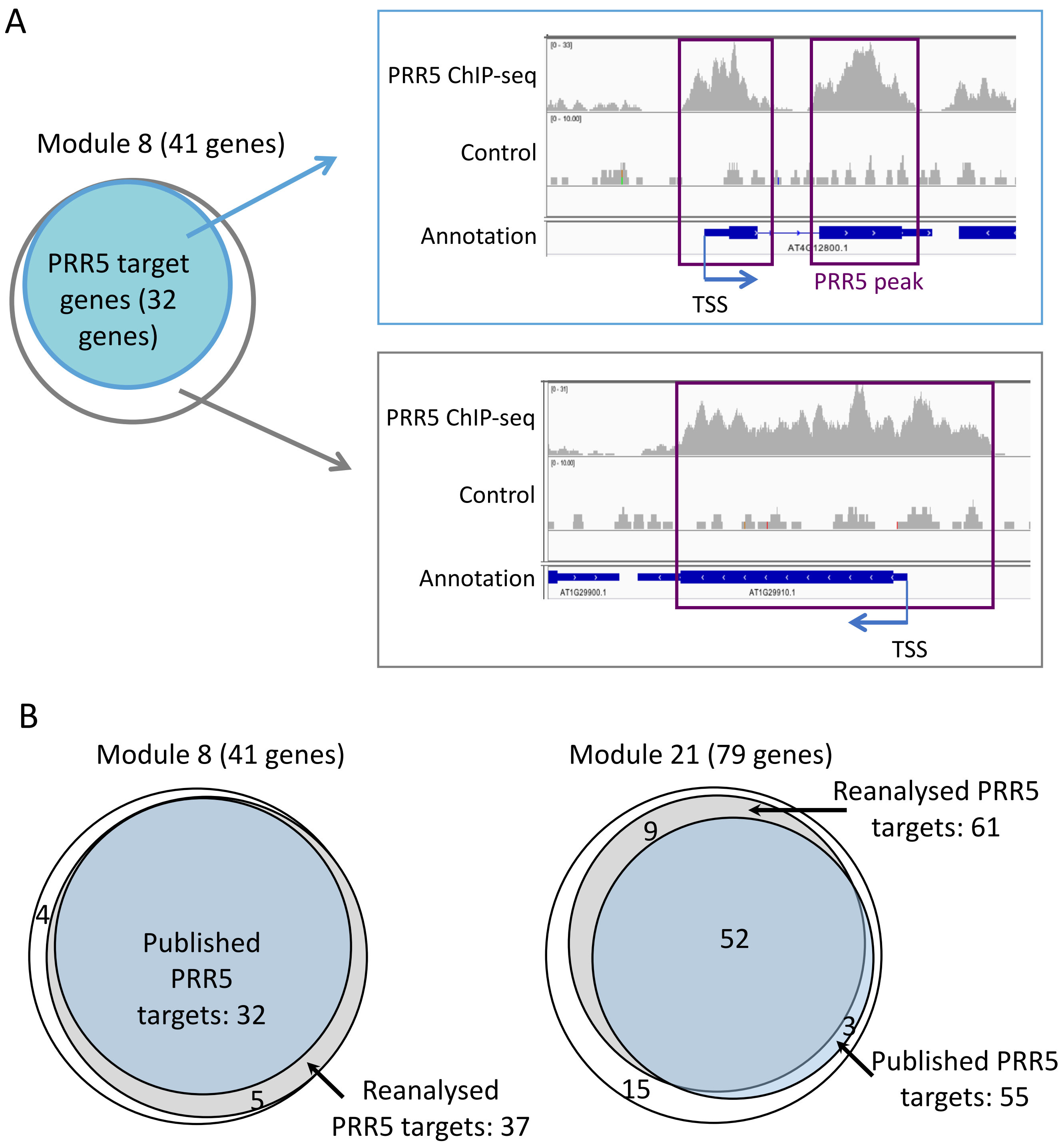

Supplementary figure 7

a. Analysis of PRR5 TF targets on the module 8: 32 of the 41 genes in the module 64 are known targets of PRR5 (left). IGV screenshot showing the signal for the PRR5 ChIP-seq (right) at a known PRR5 target (top) and for a gene in the module 64 that is not known as a PRR5 target (bottom).

b. Comparison of published (blue) and realised (grey) PRR5 targets in modules 8 (left) and 21 (right).

A

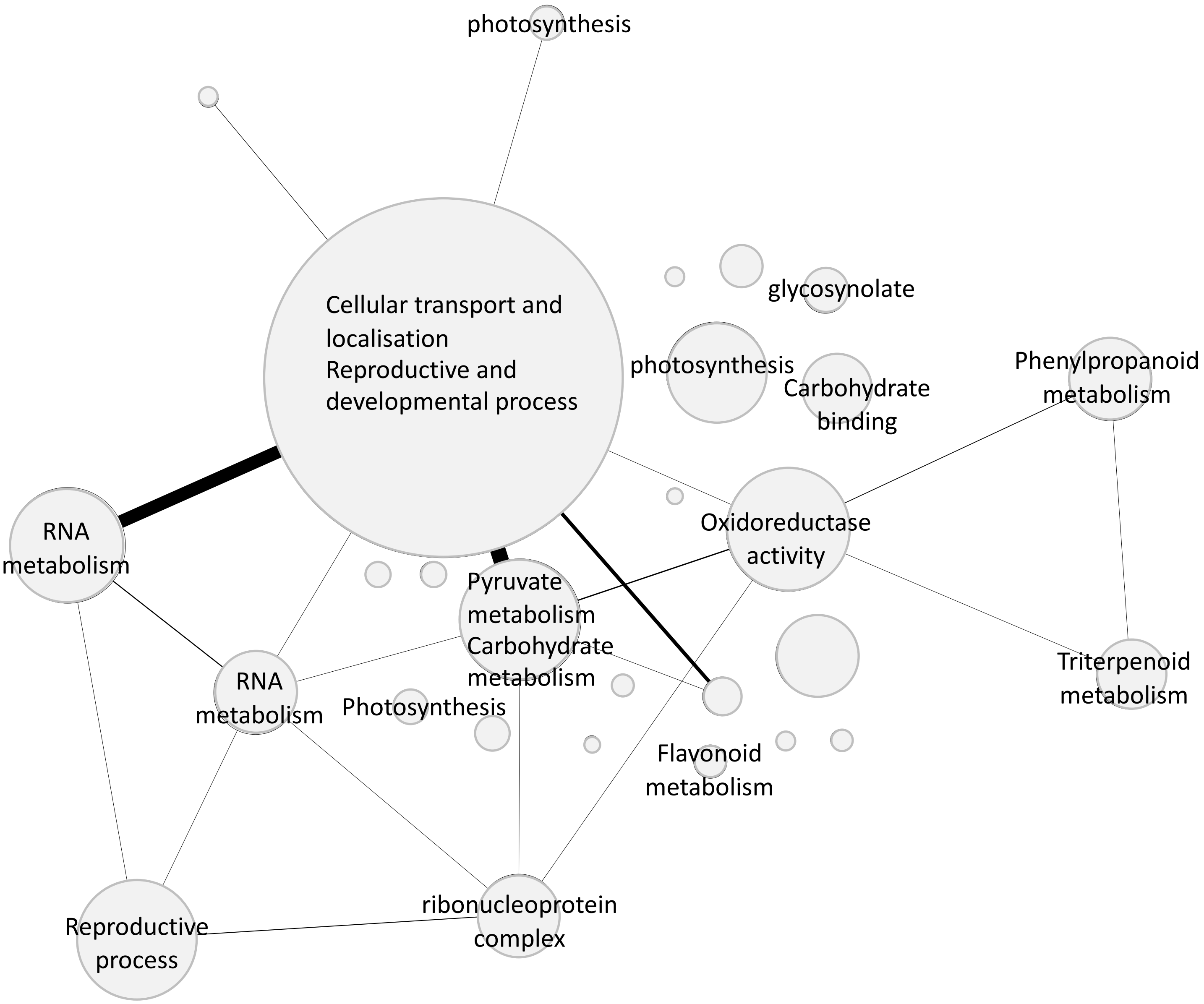
photosynthesis
Cellular transport and localisation Reproductive and developmental process
glycosynolate
photosynthesis
Carbohydrate binding
Phenylpropanoid metabolism
RNA metabolism
Oxidoreductase activity
Pyruvate metabolism Carbohydrate metabolism
RNA metabolism
Photosynthesis
Triterpenoid metabolism
Flavonoid metabolism
ribonucleoprotein complex
Reproductive process

B

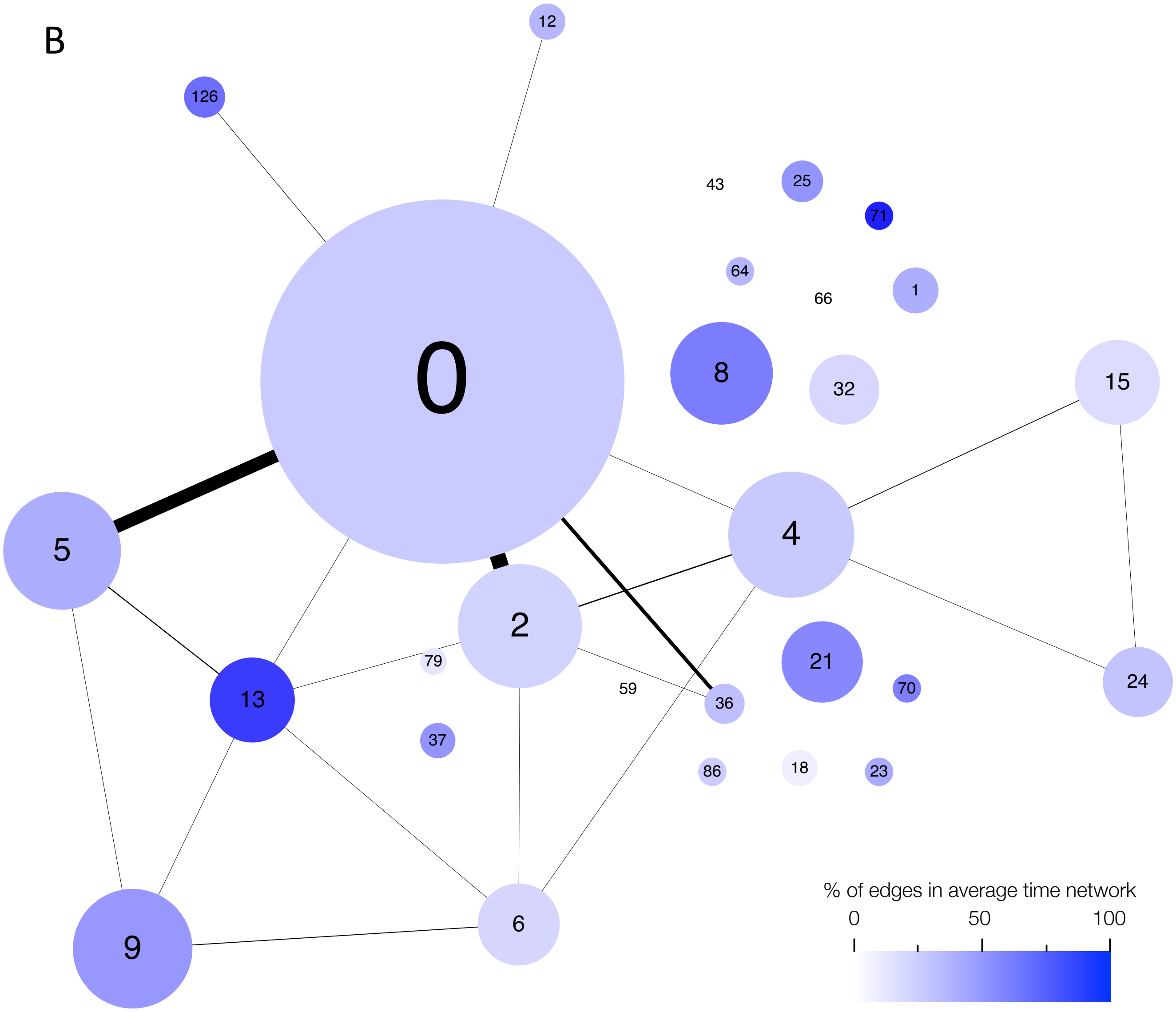

Supplementary figure 8

Organisation of modules in the network, with the size of circles representing the module size (i.e. number of edges). Number of edges connecting the modules are represented by the thickness of the lines between modules.

a. Most enriched GOs are written in each module.

b. Modules are color coded based on the percentage of edges in the averaged time course network. Dark blue modules have a high percentage of genes in the averaged time course network while light blue modules have a low percentage of genes in the averaged time course network.
